# Supplementary material for: Enhanced wind mixing and deepened mixed layer in the Pacific Arctic shelf seas with low summer sea ice
Source: Nat Commun. 2024 Nov 29;15:10389. doi: 10.1038/s41467-024-54733-w (PMC11607310; doi:10.1038/s41467-024-54733-w)
Supplement: Supplementary file 1 — Supplementary Information [file 41467_2024_54733_MOESM1_ESM.pdf]

Supplementary Information for

# Enhanced wind mixing and deepened mixed layer in the Pacific Arctic shelf seas with low summer sea ice

Yuanqi Wang[1], Zhixuan Feng[1,2*], Peigen Lin[3], Hongjun Song[4], Jicai Zhang[1], Hui Wu[1,5], Haiyan Jin[2,3,6], Jianfang Chen[2,3,6], Di Qi[7] and Jacqueline M. Grebmeier[8]

[1] State Key Laboratory of Estuarine and Coastal Research, School of Marine Sciences, and Institute of Eco-Chongming, East China Normal University, Shanghai, China

[2] State Key Laboratory of Satellite Ocean Environment Dynamics, Second Institute of Oceanography, Ministry of Natural Resources, Hangzhou, China

[3] School of Oceanography, Shanghai Jiao Tong University, Shanghai, China

[4] Observation and Research Station of Bohai Strait Eco-Corridor, First Institute of Oceanography, Ministry of Natural Resources, Qingdao, China

[5] School of Mathematical Sciences, and Key Laboratory of MEA, Ministry of Education, East China Normal University, Shanghai, China

[6] Key Laboratory of Marine Ecosystem Dynamics, Second Institute of Oceanography, Ministry of Natural Resources, Hangzhou, China

[7] Polar and Marine Research Institute, Jimei University, Xiamen, China

[8] Chesapeake Biological Laboratory, University of Maryland Center for Environmental Science, Solomons, MD, USA

[*]**Corresponding author: Z. Feng (**Email: zxfeng@sklec.ecnu.edu.cn)

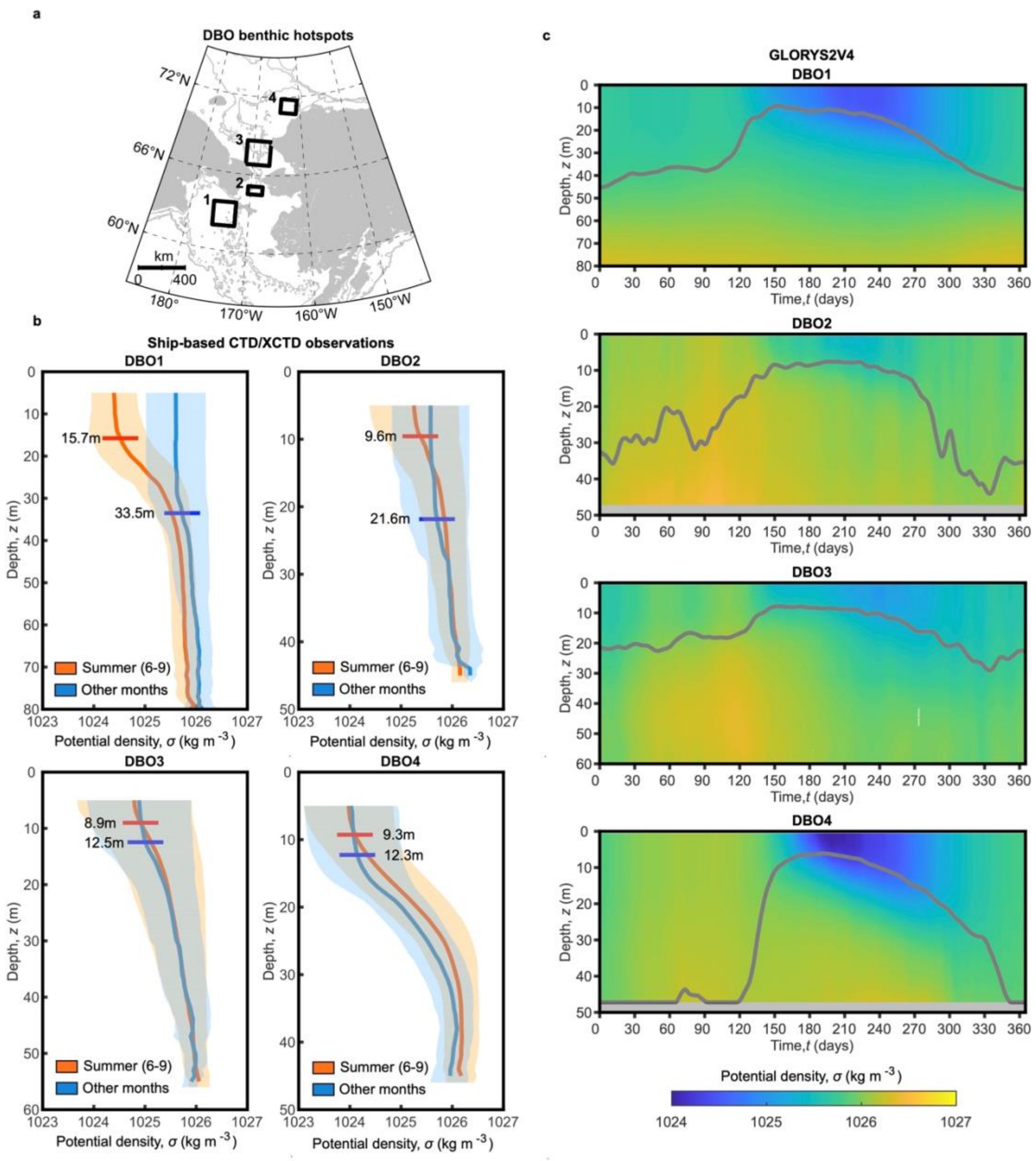


**Supplementary Fig. 1 The upper ocean structure of Distributed Biological Observatory (DBO) benthic hotspots in the Pacific Arctic Ocean. (a)** Locations of four benthic biological hotspot subdomains (black boxes) for St. Lawrence Island Polynya, Chirikov Basin, Southeastern Chukchi Sea and Northeastern Chukchi Sea under Distributed Biological Observatory framework in the northern Bering and Chukchi Seas (https://dbo.cbl.umces.edu/). Grey lines represent isobaths of 50, 250, and 500 m. **(b)** Potential density profiles in DBO regions based on the ship-based CTD/XCTD observations. The variation of mean potential density from 1996-2021 in summer (June- September) and other months is shown by orange and blue lines, respectively. The corresponding shades denote standard errors. The horizontal lines represent mixed layer depths using a threshold of $0.125\ \text{kg m}^{-3}$. **(c)** Profiles of mean potential density in DBO regions based on the GLORYS2V4. The grey lines represent the timeseries of mixed layer depth using a threshold of $0.125\ \text{kg m}^{-3}$. Source data are provided as a Source Data file.

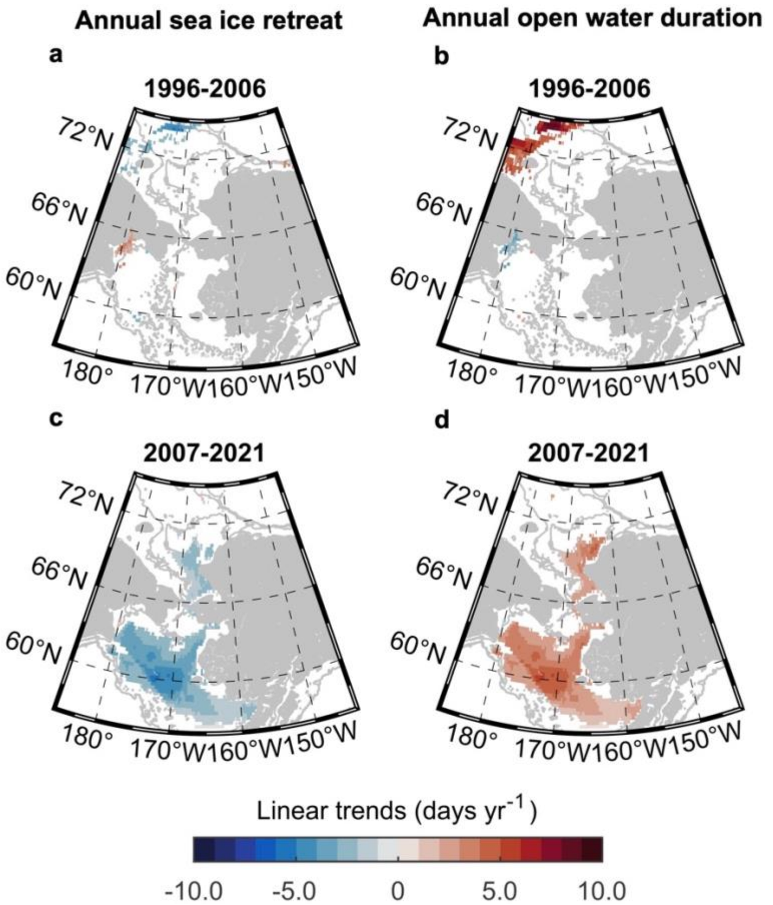


**Supplementary Fig. 2 Spatial distribution of sea-ice index trends.** Linear trends of sea ice retreat timing: **(a)** 1996-2006 and **(c)** 2007-2021. Linear trends of open water duration: **(b)** 1996-2006 and **(d)** 2007-2021. The sea ice retreat date is the day of the year when the ice concentration at each grid drops below 15% for three consecutive days after March. The start day of ice freeze is calculated as the day of the year when sea ice concentrations at each grid rise above 15% for three consecutive days. The open water (sea ice concentration < 0.15) duration is the time difference between ice retreat and freeze days. The sea-ice index trends are based on linear regression, and only significant trends with $p$-value < 0.1 are shown in blue (negative values) to red (positive values) colors. The grey lines illustrate isobath of 50 m, 250 m, and 500 m. Source data are provided as a Source Data file.

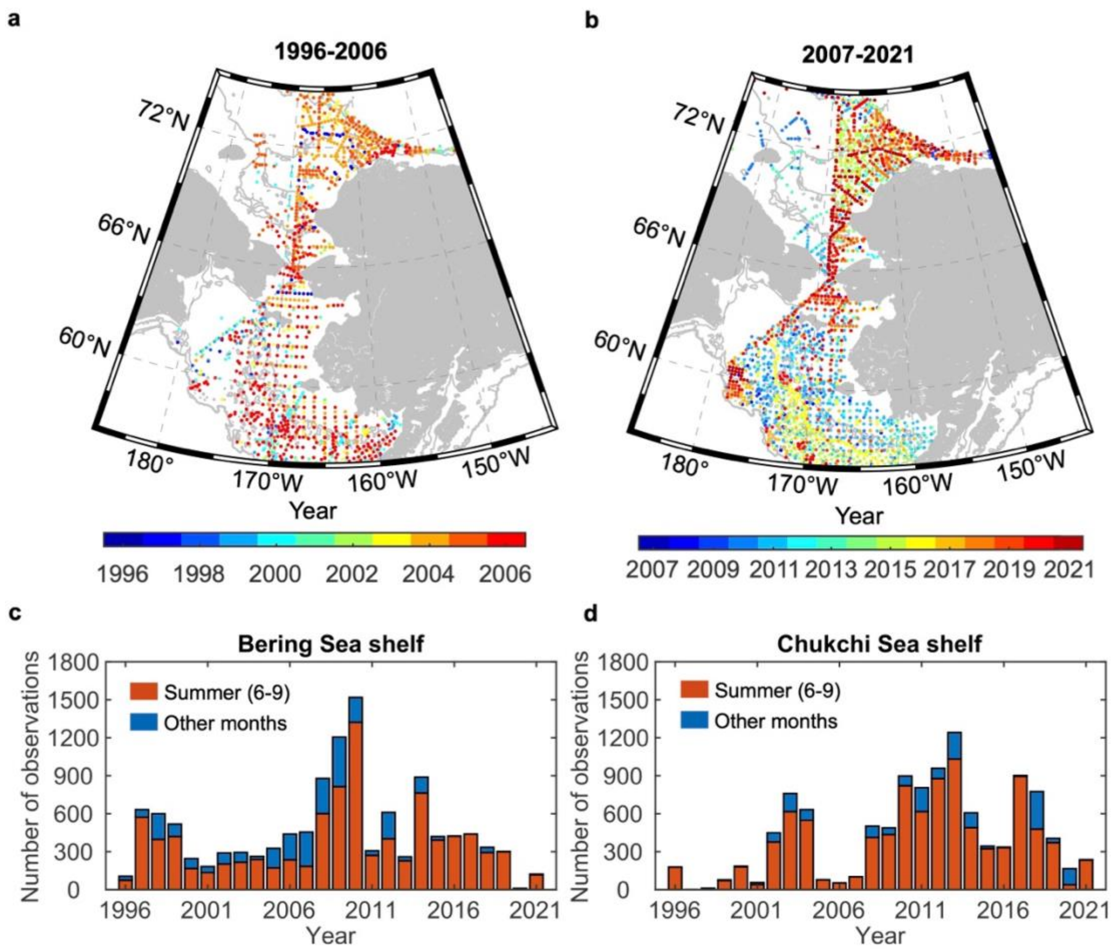


**Supplementary Fig. 3 Distribution of ship-based CTD/XCTD profiles in the study region.** Geographical locations of hydrographic profiles: **(a)** 1996-2006 and **(b)** 2007-2021 (colors of solid dots indicate the sampling years). Grey lines represent isobaths of 50, 250, and 500 m. Histograms of observations through the years and months: **(c)** Bering Sea shelf and **(d)** Chukchi Sea shelf. Total numbers of hydrographic profiles collected in the summer (June to September) and other months are shown in orange and blue bars, respectively. Source data are provided as a Source Data file.

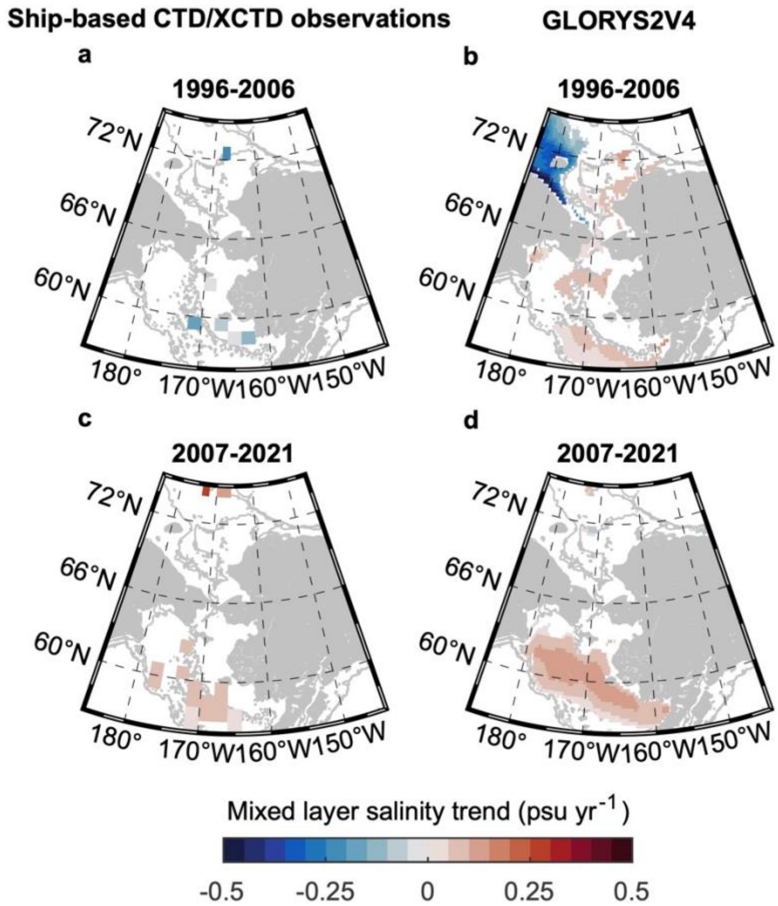


**Supplementary Fig. 4 Summer mixed layer salinity trend during different periods.** Linear trends of mean summer (June to September) mixed layer salinity (defined as the mean salinity between 5m water depth to the base of the mixed layer) in each 1°×2° grid, based on ship-based CTD/XCTD profiles during **(a)** 1996-2006 and **(c)** 2007-2021, or based on GLORYS2V4 during **(b)** 1996-2006 and **(d)** 2007-2021**.** Only linear trends with $p$-value < 0.1 are shown in blue (negative values) to red (positive values) colors. The grey lines illustrate isobath of 50 m, 250 m, and 500 m. Source data are provided as a Source Data file.

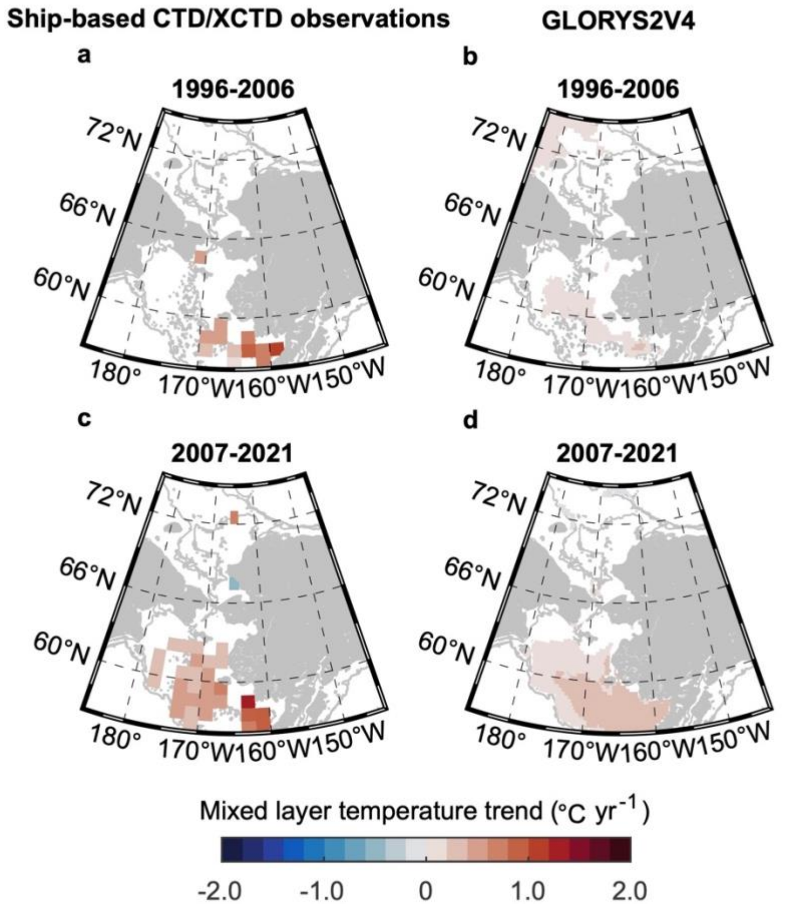


**Supplementary Fig. 5 Summer mixed layer temperature trend during different periods.** Linear trends of mean summer (June to September) mixed layer temperature (defined as the mean temperature between 5m water depth to the base of the mixed layer) in each 1°×2° grid, based on ship-based CTD/XCTD profiles during **(a)** 1996-2006 and **(c)** 2007-2021 or based on GLORYS2V4 during **(b)** 1996-2006 and **(d)** 2007-2021**.** Only linear trends with $p$-value < 0.1 are shown in blue (negative values) to red (positive values) colors. The grey lines illustrate isobath of 50 m, 250 m, and 500 m. Source data are provided as a Source Data file.

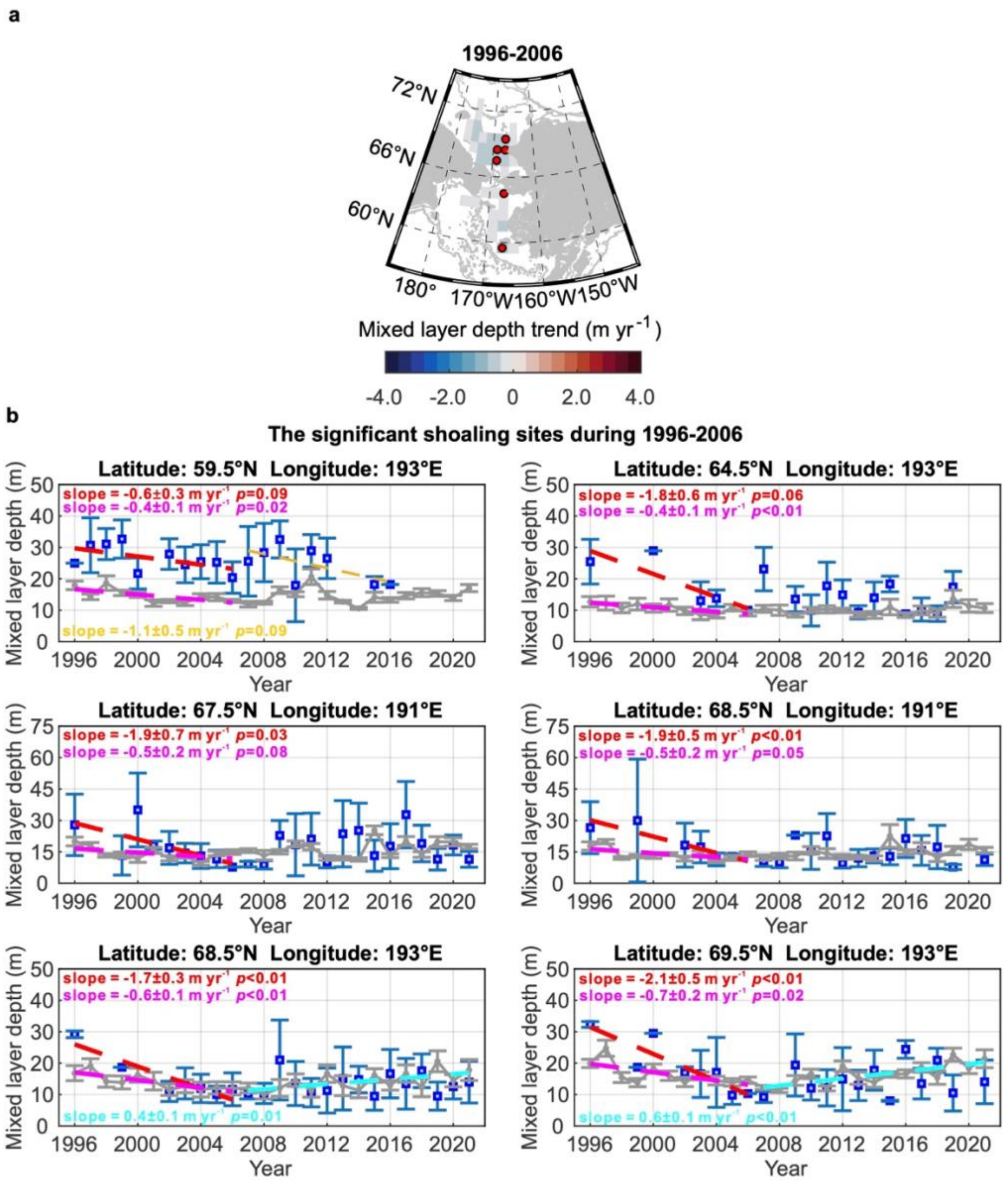


**Supplementary Fig. 6 Summer Mixed Layer Depths (MLDs) and trends at specific sites during 1996-2006. (a)** The trends of summer MLD based on Global Ocean Reanalysis and Simulations (GLORYS2V4) during 1996-2006 in each 1°×2° grid of the Pacific Arctic Ocean. Only the linear trends with *p*-value < 0.1 are shown in blue (negative values) to red (positive values) colors. Red dots are sites where both observations and model results have significant shoaling trends. The grey lines illustrate isobath of 50 m, 250 m, and 500 m. **(b)** Annual mean summer MLDs at sites corresponding to red dots. The mean summer MLDs derived from observations and GLORYS2V4 are indicated by blue squares and grey asterisks, respectively. Error bars represent the combined uncertainty from the spatiotemporal variability. The significant linear trends (*p*-value < 0.1) are shown in the dashed line. The observations during 1996-2006 are in red and in yellow during 2007-2001. The model results during 1996-2006 and 2007-2021 are in magenta and cyan lines, respectively. Source data are provided as a Source Data file.

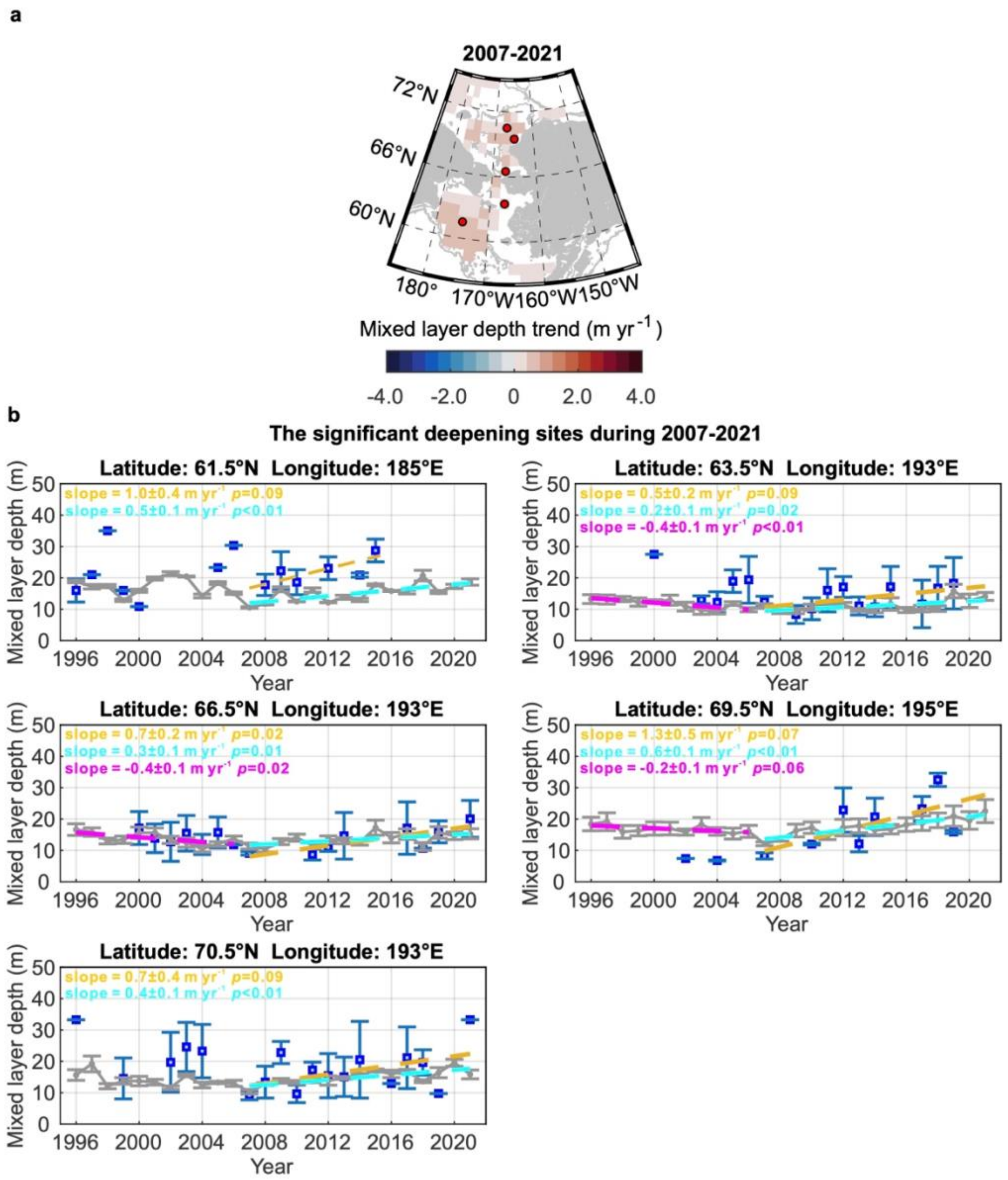


**Supplementary Fig. 7 Summer MLDs and trends at specific sites during 2007-2021.** **(a)** The trends of summer MLD based on Global Ocean Reanalysis and Simulations (GLORYS2V4) during 2007-2021 in each 1°×2° grid of the Pacific Arctic Ocean. Only the linear trends with *p*-value < 0.1 are shown in blue (negative values) to red (positive values) colors. Red dots are sites where both observations and model results have significant deepening trends. The grey lines illustrate isobath of 50 m, 250 m, and 500 m. **(b)** Annual mean summer MLDs at sites corresponding to red dots. The mean summer MLDs derived from observations and GLORYS2V4 are indicated by blue squares and grey asterisks, respectively. Error bars represent the combined uncertainty from the spatiotemporal variability. The significant linear trends (*p*-value < 0.1) are shown in the dashed line. The observations during 1996-2006 are in red and in yellow during 2007-2001. The model results during 1996-2006 and 2007-2021 are in magenta and cyan lines, respectively. Source data are provided as a Source Data file.

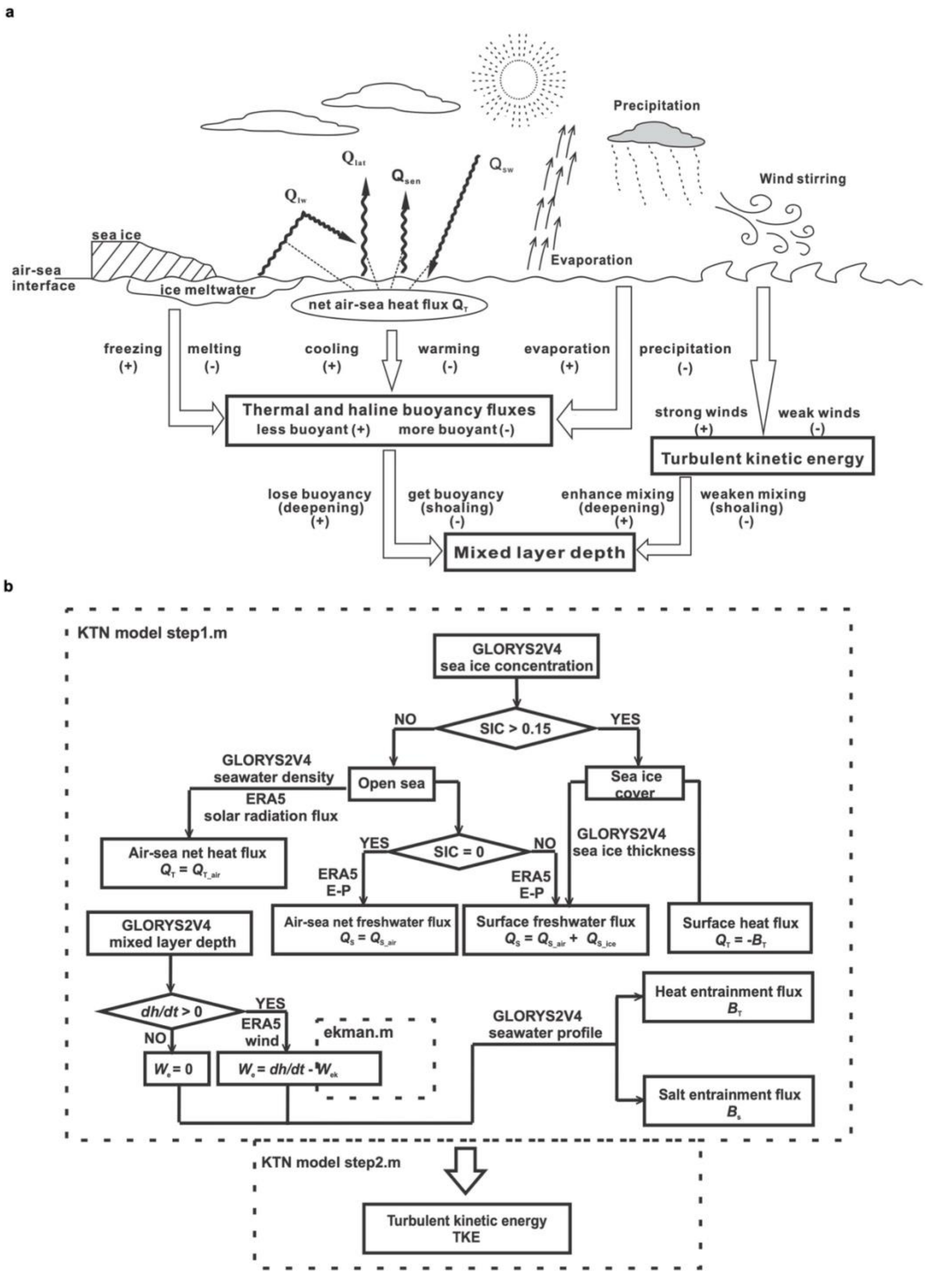


**Supplementary Fig. 8 Competing factors in 1D sea ice-ocean mixed layer model.** **(a)** Wind and surface buoyancy forces acting on the upper ocean mixed layer in open sea. Net shortwave radiation ($Q_{\mathrm{sw}}$), net longwave radiation ($Q_{\mathrm{lw}}$), latent heat flux ($Q_{\mathrm{lat}}$), and sensible heat flux ($Q_{\mathrm{sen}}$) are combined to form the net air-sea heat flux ($Q_{\mathrm{T}}$). The positive-sign factors (+) in the diagram make the upper ocean lose buoyancy and, therefore, deepen the mixed layer. The negative-sign factors (-) add buoyancy to the upper ocean and hence shoal the mixed layer. **(b)** The flow chart of 1-D sea ice-ocean mixed layer model. The dashed boxes represent three MATLAB solution codes.

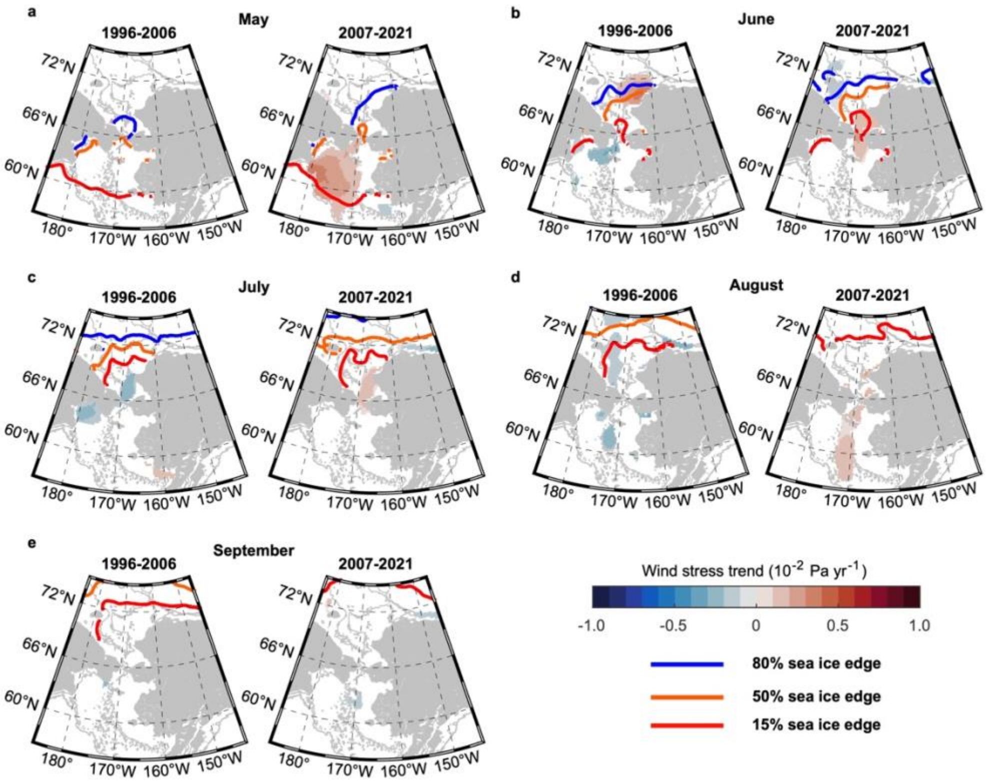


**Supplementary Fig. 9 Linear trends of monthly mean wind stress and sea ice edges during different periods.** The linear trends of wind stress in **(a)** May, **(b)** June, **(c)** July, **(d)** August, and **(e)** September during 1996-2006 and 2007-2021 in each 0.25°×0.25° grid. Only linear trends with $p$-value < 0.1 are shown in blue (negative values) to red (positive values) colors. The sea ice edges with 80%, 50%, and 15% ice concentration are indicated by blue, orange, and red lines, respectively. The grey lines illustrate isobath of 50 m, 250 m, and 500 m. Source data are provided as a Source Data file.

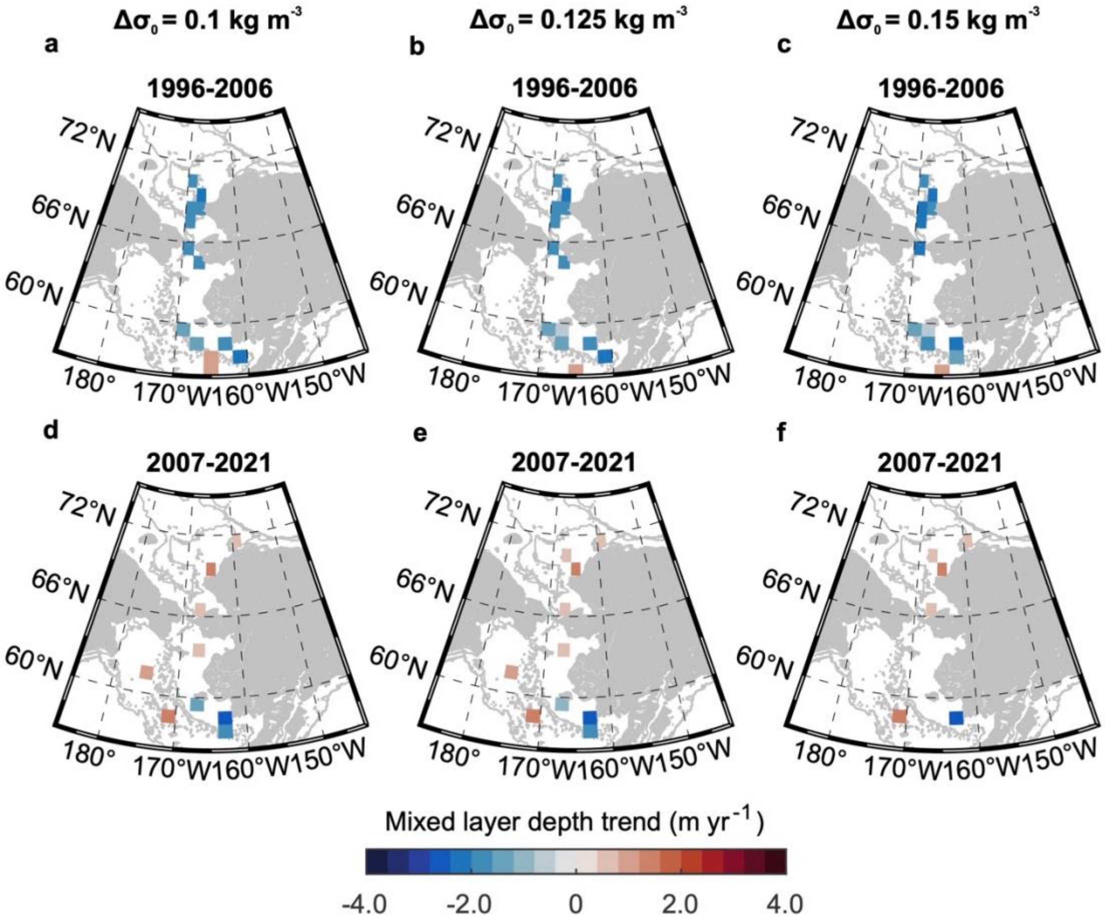


**Supplementary Fig. 10 Linear trends of the summer (June to September) MLDs based on hydrographic profiles using variable potential density thresholds of 0.1, 0.125, and 0.15 kg m$^{-3}$ during (a)-(c):** 1996-2006 and **(d)-(f):** 2007-2021. Only linear trends with *p*-value < 0.1 are shown in blue (negative values) to red (positive values) colors. Grey lines represent isobaths of 50, 250, and 500 m. Source data are provided as a Source Data file.

**Supplementary Table 1. Summary of ship-based hydrographic profiles from multinational Arctic expeditions**

| Program or cruise | Region | Expedition dates |
|---|---|---|
| Miller Freeman 1996 | Bering Sea | February-April, 1996 |
| Xue Long 1999 | Bering and Chukchi Sea | July-August, 1999 |
| Sea Storm 1999 | Bering Sea | July-September, 1999 |
| Miller Freeman 2000 | Bering Sea | February-May, 2000 |
| Sea Storm 2000 | Bering Sea | August-September, 2000 |
| RUSALCA 2000 | Chukchi Sea | August-September, 2000 |
| Miller Freeman 2001 | Bering Sea | February-June, 2001 |
| Sea Storm 2001 | Bering Sea | August-September, 2001 |
| Healy 2002 | Chukchi Sea | May-August, 2002 |
| Miller Freeman 2002 | Bering Sea | May, 2002 |
| Sea Storm 2002 | Bering Sea | August-October, 2002 |
| Miller Freeman 2003 | Bering Sea | May-September, 2003 |
| Xue Long 2003 | Bering and Chukchi Sea | July-September, 2003 |
| Sea Storm 2003 | Bering and Chukchi Sea | August-October, 2003 |
| Healy 2003 | Chukchi Sea | September–October, 2003 |
| Miller Freeman 2004 | Bering Sea | April-October, 2004 |
| Healy 2004 | Chukchi Sea | May-September, 2004 |
| RUSALCA 2004 | Chukchi Sea | August, 2004 |
| Sea Storm 2004 | Bering and Chukchi Sea | August-October, 2004 |
| Miller Freeman 2005 | Bering Sea | April-September, 2005 |
| Sea Storm 2005 | Bering and Chukchi Sea | August-October, 2005 |
| SNACS 2005 | Chukchi Sea | August – September, 2005 |
| Miller Freeman 2006 | Bering Sea | May-October, 2006 |
| Sea Storm 2006 | Bering Sea | August-September, 2006 |
| Miller Freeman 2007 | Bering Sea | April-May, 2007 |
| Sea Storm 2007 | Bering Sea | August-October, 2007 |

| | | |
|---|---|---|
| Northwest Explorer 2007 | Bering and Chukchi Sea | September, 2007 |
| Healy 2008 | Bering and Chukchi Sea | March -September, 2008 |
| Oscar Dyson 2008 | Bering Sea | May, 2008 |
| BSIERP 2008 | Bering Sea | June -July, 2008 |
| Xue Long 2008 | Bering and Chukchi Sea | July-September, 2008 |
| Miller Freeman 2008 | Bering Sea | August-September, 2008 |
| MIRAI MR08-04 | Chukchi Sea | August – October, 2008 |
| Oscar Dyson 2009 | Bering Sea | March-May, 2009 |
| Healy 2009 | Bering and Chukchi Sea | March -May, 2009 |
| | Chukchi Sea | July-August, 2009 |
| IPHC 2009 | Bering Sea | May – July, 2009 |
| BSIERP 2009 | Bering Sea | June -July, 2009 |
| Knorr 2009 | Bering Sea | June -July, 2009 |
| Point Sur 2009 | Bering Sea | July, 2009 |
| Sea Storm 2009 | Bering Sea | August-September, 2009 |
| RUSALCA 2009 | Chukchi Sea | September, 2009 |
| Epic Explorer 2009 | Bering Sea | September, 2009 |
| Miller Freeman 2009 | Bering Sea | September-October, 2009 |
| Louis S. St-Laurent 2009 | Chukchi Sea | September - October, 2009 |
| Polar Sea 2010 | Bering Sea | March -April, 2010 |
| Thompson 2010 | Bering Sea | May-July, 2010 |
| BSIERP 2010 | Bering Sea | June-August, 2010 |
| Healy 2010 | Chukchi Sea | June-July, 2010 |
| | | September, 2010 |
| IPHC 2010 | Bering Sea | June-August, 2010 |
| Point Sur 2010 | Bering Sea | July-August, 2010 |
| Xue Long 2010 | Bering and Chukchi Sea | July-August, 2010 |
| Alaskan Enterprise 2010 | Chukchi Sea | August-September, 2010 |
| Annika Marie 2010 | Chukchi Sea | August-September, 2010 |

| | | |
|---|---|---|
| Wecoma 2010 | Bering Sea | August-September, 2010 |
| Great Pacific 2010 | Bering Sea | August-September, 2010 |
| Sea Storm 2010 | Bering Sea | September-October, 2010 |
| Louis S. St-Laurent 2010 | Chukchi Sea | September-October, 2010 |
| Westward Wind 2010 | Chukchi Sea | August-October, 2010 |
| IPHC 2011 | Bering Sea | June-August, 2011 |
| Healy 2011 | Chukchi Sea | June-December, 2011 |
| Sir Wilfrid Laurier 2011 | Chukchi Sea | July, 2011 |
| Louis S. St-Laurent 2011 | Chukchi Sea | July-August, 2011 |
| Westward Wind 2011 | Chukchi Sea | August-September, 2011 |
| Mystery Bay 2011 | Bering and Chukchi Sea | August-September, 2011 |
| Sea Storm 2011 | Bering Sea | August-September, 2011 |
| Annika Marie 2011 | Chukchi Sea | August-September, 2011 |
| Great Pacific 2011 | Bering Sea | August-September, 2011 |
| Oscar Dyson 2011 | Bering Sea | September, 2011 |
| Oscar Dyson 2012 | Bering Sea | April-October, 2012 |
| IPHC 2012 | Bering Sea | May-July, 2012 |
| Sir Wilfrid Laurier 2012 | Chukchi Sea | July, 2012 |
| Xue Long 2012 | Bering and Chukchi Sea | July-September, 2012 |
| Louis S. St-Laurent 2012 | Chukchi Sea | August-September, 2012 |
| Bristol Explorer 2012 | Bering and Chukchi Sea | August-September, 2012 |
| Fairweather 2012 | Chukchi Sea | August, 2012 |
| Aquila 2012 | Bering and Chukchi Sea | August-September, 2012 |
| Sea Storm 2012 | Bering and Chukchi Sea | August-September, 2012 |
| Healy 2012 | Chukchi Sea | August-October, 2012 |
| Annika Marie 2012 | Chukchi Sea | August-September, 2012 |
| Miller Freeman 2012 | Bering Sea | September, 2012 |
| MIRAI MR12-03 | Chukchi Sea | September-October, 2012 |
| Oscar Dyson 2013 | Bering Sea | May, 2013 |

| | | |
|---|---|---|
| IPHC 2013 | Bering Sea | June-August, 2013 |
| Sir Wilfrid Laurier 2013 | Chukchi Sea | July, 2013 |
| Louis S. St-Laurent 2013 | Chukchi Sea | August, 2013 |
| Healy 2013 | Chukchi Sea | August-October, 2013 |
| Sea Storm 2013 | Bering and Chukchi Sea | August-September, 2013 |
| Annika Marie 2013 | Chukchi Sea | August-September, 2013 |
| MIRAI MR13-06 | Bering and Chukchi Sea | August-October, 2013 |
| Bristol Explorer 2013 | Chukchi Sea | August-September, 2013 |
| Healy 2014 | Chukchi Sea | May-August, 2014 |
| IPHC 2014 | Bering Sea | June-August, 2014 |
| Norseman 2014 | Chukchi Sea | July, 2014 |
| Sir Wilfrid Laurier 2014 | Chukchi Sea | July, 2014 |
| Xue Long 2014 | Bering and Chukchi Sea | June-August, 2014 |
| Bristol Explorer 2014 | Bering Sea | August-October, 2014 |
| Annika Marie 2014 | Chukchi Sea | August-September, 2014 |
| Sea Storm 2014 | Bering Sea | September, 2014 |
| Alaska Endeavor 2014 | Bering Sea | September, 2014 |
| Oscar Dyson 2014 | Bering Sea | May-October, 2014 |
| Aquila 2014 | Chukchi Sea | September-October, 2014 |
| MIRAI MR14-05 | Bering and Chukchi Sea | September, 2014 |
| CCGS Louis S. St-Laurent 2014 | Chukchi Sea | September-October, 2014 |
| Sikuliaq 2015 | Bering and Chukchi Sea | March - October, 2015 |
| Norseman 2015 | Bering Sea | July, 2015 |
| IPHC 2015 | Bering Sea | June-August, 2015 |
| Sir Wilfrid Laurier 2015 | Chukchi Sea | July, 2015 |
| Sea Storm 2015 | Bering Sea | September, 2015 |
| MIRAI MR15-03 | Chukchi Sea | September-October, 2015 |
| Bristol Explorer 2015 | Bering Sea | September-October, 2015 |
| Louis S. St-Laurent 2015 | Chukchi Sea | September-October, 2015 |

| | | |
|---|---|---|
| IPHC 2016 | Bering Sea | June-August, 2016 |
| Norseman 2016 | Chukchi Sea | July, 2016 |
| Healy 2016 | Bering and Chukchi Sea | July-August, 2016 |
| Xue Long 2016 | Bering and Chukchi Sea | July-September, 2016 |
| Alaskan Endeavor 2016 | Bering Sea | August-September, 2016 |
| Sea Storm 2016 | Bering Sea | August-September, 2016 |
| MIRAI MR16-06 | Chukchi Sea | August-September, 2016 |
| Sikuliaq 2016 | Chukchi Sea | September, 2016 |
| Louis S. St-Laurent 2016 | Chukchi Sea | September-October, 2016 |
| Sikuliaq 2017 | Bering and Chukchi Sea | June-September, 2017 |
| IPHC 2017 | Bering Sea | June-August, 2017 |
| Norseman 2017 | Chukchi Sea | July, 2017 |
| Oshoro Maru 2017 | Chukchi Sea | July, 2017 |
| Healy 2017 | Bering and Chukchi Sea | July-October, 2017 |
| Xue Long 2017 | Bering and Chukchi Sea | July-September, 2017 |
| Araon 2017 | Chukchi Sea | August, 2017 |
| Ocean Starr 2017 | Chukchi Sea | August-September, 2017 |
| Epic Explorer 2017 | Bering Sea | August-September, 2017 |
| MIRAI MR17-05 | Bering and Chukchi Sea | August-September, 2017 |
| Louis S. St-Laurent 2017 | Chukchi Sea | September-October, 2017 |
| Sikuliaq 2018 | Bering and Chukchi Sea | June-October, 2018 |
| Oshoro Maru 2018 | Bering Sea | July, 2018 |
| IPHC 2018 | Bering Sea | July-August,2018 |
| Araon 2018 | Chukchi Sea | August, 2018 |
| Healy 2018 | Chukchi Sea | August-November, 2018 |
| Norseman 2018 | Chukchi Sea | August, 2018 |
| Xue Long 2018 | Chukchi Sea | August-September, 2018 |
| Sea Storm 2018 | Bering and Chukchi Sea | August-September, 2018 |
| Louis S. St-Laurent 2018 | Chukchi Sea | September, 2018 |

| | | |
|---|---|---|
| Northwest Explorer 2018 | Chukchi Sea | September-October, 2018 |
| MIRAI MR18-05 | Bering and Chukchi Sea | October-November, 2018 |
| IPHC 2019 | Bering Sea | July-September,2019 |
| Araon 2019 | Bering and Chukchi Sea | August, 2019 |
| Healy 2019 | Chukchi Sea | August-September, 2019 |
| Xue Long 2019 | Bering and Chukchi Sea | August-September, 2019 |
| Sea Storm 2019 | Bering Sea | August-September, 2019 |
| Norseman 2019 | Chukchi Sea | September, 2019 |
| Louis S. St-Laurent 2019 | Chukchi Sea | September-October, 2019 |
| MIRAI MR19-03 | Bering and Chukchi Sea | October-November, 2019 |
| Sikuliaq 2019 | Chukchi Sea | November, 2019 |
| Araon 2020 | Chukchi Sea | August, 2020 |
| Xue Long 2020 | Bering and Chukchi Sea | July-September, 2020 |
| Louis S. St-Laurent 2020 | Chukchi Sea | September-October, 2020 |
| MIRAI MR20-05 | Chukchi Sea | October, 2020 |
| Sikuliaq 2020 | Bering and Chukchi Sea | October-November, 2020 |
| IPHC 2021 | Bering Sea | June-July, 2021 |
| Norseman 2021 | Chukchi Sea | July, 2021 |
| Louis S. St-Laurent 2021 | Chukchi Sea | August-September, 2021 |
| MIRAI MR21-05 | Chukchi Sea | September-October, 2020 |

**Supplementary Table 2. Summary of ship-based hydrographic profiles extracted from the World Ocean Database 2018**

| Program or cruise | Region | Expedition dates |
|---|---|---|
| Oshoro Maru 1996 | Bering Sea | July-August, 1996 |
| Miller Freeman 1996 | Bering Sea | July-September, 1996 |
| Kaiyo Maru 1996 | Bering Sea | August-October, 1996 |
| R.B.YOUNG 1996 | Chukchi Sea | September, 1996 |
| Alpha Helix 1996 | Chukchi Sea | September, 1996 |
| Miller Freeman 1997 | Bering Sea | February-May, 1997<br>July-September, 1997 |
| Wecoma 1997 | Bering Sea | June-July, 1997 |
| Alpha Helix 1997 | Bering Sea | June, 1997<br>August-September, 1997 |
| Hakuho Maru 1997 | Bering Sea | July, 1997 |
| Oshoro Maru 1997 | Bering Sea | July-August, 1997 |
| Louis S. St-Laurent 1997 | Chukchi Sea | September, 1997 |
| Miller Freeman 1998 | Bering Sea | February-April, 1998 |
| Wecoma 1998 | Bering Sea | May-June, 1998 |
| Alpha Helix 1998 | Bering Sea | May-June, 1998<br>August-September, 1998 |
| Oshoro Maru 1998 | Bering Sea | July-August, 1998 |
| Sir Wilfrid Laurier 1998 | Bering Sea | September-October, 1998 |
| Thomas G. Thompson 1999 | Bering Sea | February, 1999 |
| Miller Freeman 1999 | Bering and Chukchi Sea | April-May, 1999<br>July-September, 1999 |
| Wecoma 1999 | Bering Sea | May, 1999 |
| Oshoro Maru 1999 | Bering Sea | July-August, 1999 |
| Alpha Helix 1999 | Bering Sea | July-August, 1999 |
| Sir Wilfrid Laurier 1999 | Bering and Chukchi Sea | September-October, 1999 |
| MIRAI 1999 | Chukchi Sea | September, 1999 |

| | | |
|---|---|---|
| Miller Freeman 2000 | Bering Sea | June-August, 2000 |
| Oshoro Maru 2000 | Bering Sea | July-August, 2000 |
| Sir Wilfrid Laurier 2000 | Bering and Chukchi Sea | July-October, 2000 |
| Alpha Helix 2000 | Bering Sea | August-September, 2000 |
| MIRAI MR00-K06 | Bering Sea | September, 2000 |
| Sir Wilfrid Laurier 2001 | Bering and Chukchi Sea | July-October,2001 |
| Oshoro Maru 2001 | Bering Sea | July-August, 2001 |
| MIRAI 2001 | Bering Sea | August, 2001 |
| Alpha Helix 2001 | Bering and Chukchi Sea | September, 2001 |
| Miller Freeman 2002 | Bering Sea | April-March, 2002 |
| Alpha Helix 2002 | Bering and Chukchi Sea | June, 2002 |
| Ocean Explorer 2002 | Bering Sea | June-July, 2002 |
| Polar Star 2002 | Chukchi Sea | July-August, 2002 |
| Sir Wilfrid Laurier 2002 | Bering and Chukchi Sea | July-September, 2002 |
| Louis S. St-Laurent 2002 | Chukchi Sea | August-September, 2002 |
| MIRAI MR02-K05 | Bering and Chukchi Sea | September-October, 2002 |
| Miller Freeman 2003 | Bering Sea | March, 2003 |
| Sir Wilfrid Laurier 2003 | Bering and Chukchi Sea | July, 2003 |
| Alpha Helix 2003 | Bering and Chukchi Sea | July, 2003 |
| Nathaniel B. Palmer 2003 | Chukchi Sea | July-August, 2003 |
| Louis S. St-Laurent 2003 | Chukchi Sea | August-September, 2003 |
| Sir Wilfrid Laurier 2004 | Bering and Chukchi Sea | July, 2004 |
| MIRAI MR04-K05 | Bering Sea | August, 2004 |
| Louis S. St-Laurent 2004 | Chukchi Sea | August, 2004 |
| Alpha Helix 2004 | Bering and Chukchi Sea | August-September, 2004 |
| Healy 2005 | Chukchi Sea | June-July, 2005 |
| Sir Wilfrid Laurier 2005 | Bering and Chukchi Sea | July, 2005 |
| Louis S. St-Laurent 2005 | Chukchi Sea | August, 2005 |
| RUSALCA 2005 | Bering Sea | August, 2005 |

| | | |
|---|---|---|
| Oden 2005 | Chukchi Sea | August-September, 2005 |
| Amundsen 2005 | Chukchi Sea | September, 2005 |
| RUSALCA 2006 | Bering Sea | August, 2006 |
| MIRAI 2006 | Bering and Chukchi Sea | August-September, 2006 |
| Louis S. St-Laurent 2006 | Chukchi Sea | August-September, 2006 |
| Healy 2007 | Bering Sea | April-May, 2007 |
| Louis S. St-Laurent 2007 | Chukchi Sea | July-August, 2007 |
| RUSALCA 2007 | Chukchi Sea | September, 2007 |
| MIRAI 2007 | Bering Sea | October, 2007 |
| Oshoro Maru 2008 | Bering and Chukchi Sea | June-July, 2008 |
| Rainier 2008 | Bering Sea | July-August, 2008 |
| Louis S. St-Laurent 2008 | Chukchi Sea | July-August, 2008 |
| Bluefin 2008 | Chukchi Sea | July-October, 2008 |
| Ocean Explorer 2008 | Chukchi Sea | August, 2008 |
| Amundsen 2009 | Chukchi Sea | July-August, 2009 |
| Westward Wind 2009 | Chukchi Sea | August-September, 2009 |
| MIRAI MR09-03 | Chukchi Sea | September-October, 2009 |
| Fairweather 2010 | Bering Sea | July-September, 2010 |
| RUSALCA 2010 | Bering and Chukchi Sea | August, 2010 |
| MIRAI MR10-05 | Bering Sea | October-November, 2010 |
| RUSALCA 2011 | Bering and Chukchi Sea | August, 2011 |
| Fairweather 2011 | Bering Sea | July-October, 2011 |
| RUSALCA 2012 | Bering and Chukchi Sea | July-September, 2012 |
| Frosti 2012 | Chukchi Sea | August-September, 2012 |
| Westward Wind 2012 | Chukchi Sea | August-October, 2012 |
| Oshoro Maru 2013 | Bering and Chukchi Sea | June-July, 2013 |
| Norseman 2013 | Bering and Chukchi Sea | July-August, 2013 |
| Frosti 2013 | Chukchi Sea | August-September, 2013 |
| Westward Wind 2013 | Chukchi Sea | August-October, 2013 |

| | | |
|---|---|---|
| Tara 2013 | Chukchi Sea | September, 2013 |
| Rainier 2014 | Bering Sea | July, 2014 |
| Akademik Tryoshnikov 2015 | Chukchi Sea | August-September, 2015 |
| Healy 2015 | Bering and Chukchi Sea | August-October, 2015 |
